# The association between oral health problems and malnutrition in a population-based sample of older adults – The HUNT Study

**DOI:** 10.1016/j.jnha.2026.100935

**Published:** 2026-08-07

**Authors:** Sandra H. Cetrelli, Yi-Qian Sun, Ingvild Paur, Hedda Høvik, Tone N. Fagerhaug, Lars Martin Berg, Håvard K. Skjellegrind, Wenche M. Thorstensen, Marit Kolberg

**Affiliations:** a Department of Neuromedicine and Movement Science, Norwegian University of Science and Technology, Trondheim, Norway; b Center for Oral Health Services and Research, Mid-Norway (TkMidt), Trondheim, Norway; c Department of Clinical and Molecular Medicine, Norwegian University of Science and Technology, Trondheim, Norway; d Department of Pathology, Clinic of Laboratory Medicine, St. Olavs Hospital, Trondheim University Hospital, Norway; e Norwegian National Network for Disease-Related Malnutrition, Oslo, Norway; f Section for Clinical Nutrition, Department of Clinical Services, Division of Cancer Medicine, Oslo University Hospital, Oslo, Norway; g Department of Clinical Medicine, Clinical Nutrition Research Group, UiT The Arctic University of Norway, Tromsø, Norway; h Department of Public Health and Nursing, Norwegian University of Science and Technology, Trondheim, Norway; i Department of Clinical Dentistry, UiT The Arctic University of Norway, Tromsø, Norway; j HUNT Research Centre, Department of Public Health and Nursing, Norwegian University of Science and Technology, Levanger, Norway; k Levanger Hospital, Nord-Trøndelag Hospital Trust, Levanger, Norway; l Department of Otolaryngology, Head and Neck Surgery, St. Olavs Hospital, Trondheim University Hospital, Norway

**Keywords:** HUNT, HUNT4 70+, Older adults, Oral health, Malnutrition

## Abstract

**Objective:**

Investigate the relationship between oral health problems and malnutrition among Norwegian older adults.

**Design:**

Cross-sectional study.

**Setting and participants:**

We used data from 1571 participants in HUNT4 70+, a sub-study on participants ≥70 years of age within the fourth Trøndelag Health Study (HUNT4).

**Measurements:**

The Revised Oral Assessment Guide-Jönköping (ROAG-J) was used to evaluate individual oral health items and to compute a total score and an ordinal variable for problem severities. The number of teeth and denture use were also included. Malnutrition was determined using body mass index, self-reported involuntary weight loss, and food intake. Poisson regression with robust error variance was used to estimate the association between oral health problems and the prevalence of malnutrition presented as prevalence ratios (PR) with 95% confidence intervals (CIs).

**Results:**

The median age was 76.9 years, with 57.6% being women. A total of 57.5% of participants had oral health problems, and 17.8% had malnutrition. An increase in ROAG-J total score (PR 1.02, 95% CI 1.01–1.03) and severe oral health problems (PR 1.09, 95% CI 1.03–1.15) were associated with a higher prevalence of malnutrition. For individual ROAG-J items, problems with voice, lips, and dentures were associated with a higher prevalence of malnutrition.

**Conclusion:**

Both the number and severity of oral health problems were associated with the prevalence of malnutrition. Our findings suggest that efforts should be made to prevent and address both oral health problems and malnutrition among Norwegian older adults.

## 1 Introduction

Malnutrition is a major health challenge in the increasing population of older adults, especially among the ill and the care-dependent [[1], [2], [3]]. Malnutrition is associated with several negative outcomes among older adults, e.g., decreased quality of life, cognitive impairment, poorer recovery from illness, increasing health care costs, and shorter survival [1]. This underscores the importance of preventing malnutrition for maintaining good health. It is increasingly evident that the cause of malnutrition is multifaceted [1]. Oral health is an important part of general health and contributes to quality of life [4]. It has been proposed that oral health problems, such as poor dental status, oral pain, dry mouth, and impaired chewing, alongside other health and demographic variables, may directly or indirectly cause malnutrition [5].

When identifying potential causes of malnutrition, a dental examination or oral health assessment should be considered [6]. Before selecting an appropriate intervention, chewing and swallowing abilities should be evaluated [6]. Recent systematic reviews suggest that denture use, number of teeth (including tooth loss and edentulism), and chewing difficulties are associated with reduced nutrient and food intake [7,8]. For malnutrition risk assessed by screening tools, studies suggest that a non-functional dentition, but not denture use, is associated with the risk of malnutrition [9,10]. Furthermore, for individual measures of malnutrition, such as weight loss and low body mass index, there are limited or conflicting findings regarding edentulism, tooth loss, denture use, issues with gums, swallowing and chewing problems, and oral pain [11]. Varying findings can partially be explained by differences in the methods used to measure both oral health and malnutrition.

Oral health measures are often assessed through self-reported data in larger population studies investigating the association between oral health and malnutrition [[12], [13], [14]]. Screening tools that provide standardised measures of oral health can provide valuable insight into the oral health status of older adults [15]. Investigating the relationship between standardised measures of oral health problems and malnutrition can further strengthen the evidence. Additionally, no studies have addressed the association in population-based settings, including both care-independent and care-dependent older individuals in Norway. Thus, the aim of this study was to assess the association between oral health problems and the prevalence of malnutrition in a population-based sample of older adults.

## 2 Methods

### 2.1 Study population

The current study is based on data from a cohort of adults aged ≥70 years who participated in the fourth wave of the Trøndelag Health Study (HUNT4), Norway [16]. This specific data collection among older adults is referred to as HUNT4 70+ and consists of two sub-cohorts: HUNT4 70+ and HUNT4 Trondheim 70+ [17]. HUNT4 70+ was conducted between August 2017 and February 2019 in 23 municipalities in the northern part of Trøndelag County. HUNT4 Trondheim 70+ was conducted between October 2018 and June 2019, and included the eastern districts of Trondheim, the largest city in Trøndelag County [17]. All adult inhabitants in Trøndelag County were invited by mail to participate in HUNT4. The study population, including HUNT4 70+, is considered representative of the Norwegian population [16].

Participants completed self-report questionnaires and attended standardised interviews and physical assessments. In total, 11 699 participated in the 70+ cohorts. Three types of test locations were used for data collection: field stations, home visits, and nursing homes. One field station was established in each municipality. For home visits and nursing homes, ambulant teams collected data from participants who were unable to attend field stations. The majority of participants examined during home visits were recruited through their local home-care services. The data collection personnel consisted of local health care workers and nursing students. Similar data collection procedures were applied in both sub-cohorts and at all three test locations, except for the oral health assessment. In the HUNT4 70+ cohort, the oral health assessment was only performed at home visits and in nursing homes, not at the field stations. In HUNT4 Trondheim 70+, the oral health assessment was performed at all test locations [17].

**Fig. 1:**
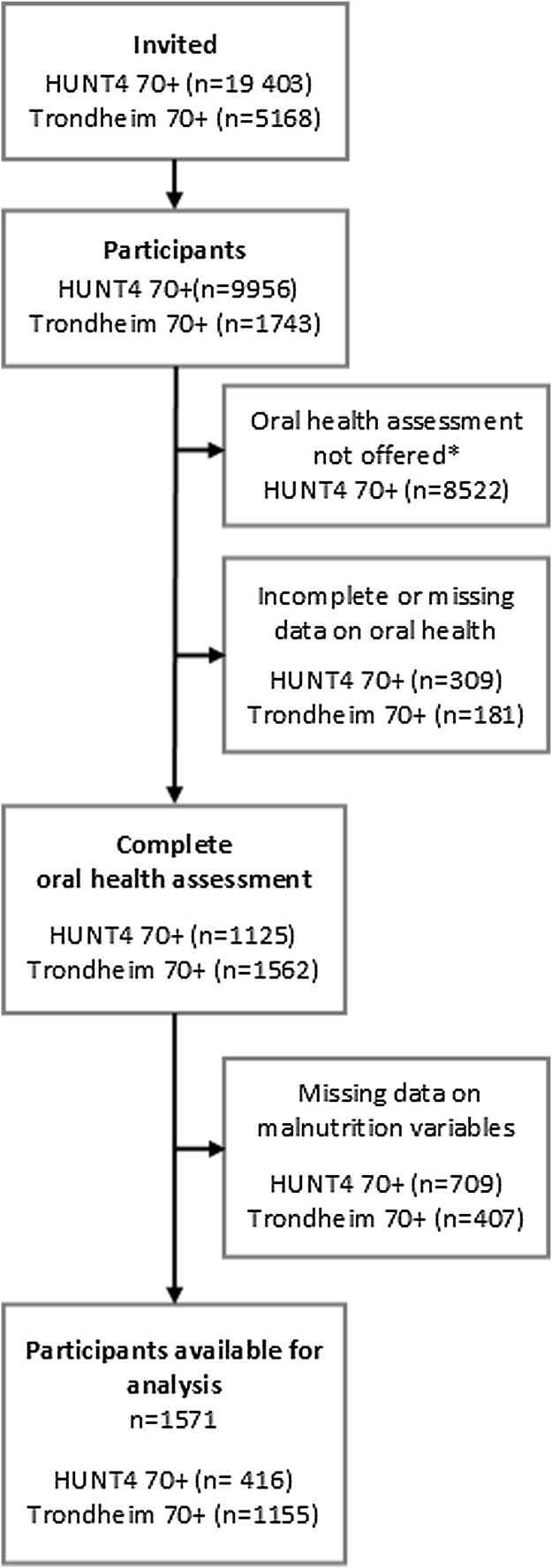
Selection of study participants. The final study sample had complete data on the oral health assessment (ROAG-J) and malnutrition variables (body mass index, involuntary weight loss, and food intake). *Participants in field stations in HUNT4 70+ were not offered the oral health assessment.

Participants from both sub-cohorts were included if they had complete data on the oral health assessment and on selected malnutrition variables. Fig. 1 shows a flow chart of the selection of study participants.

### 2.2 Ethics

Participation in HUNT4 was voluntary. Written informed consent was obtained from all participants or from the next of kin. The HUNT Study was approved by the Norwegian Data Protection Authority. Approval to conduct the current study was obtained from the Regional Committee for Medical and Health Research Ethics in Norway (ref. 81406) and the Norwegian Agency for Shared Services in Education and Research (ref. 154423). The handling of data was performed in accordance with established guidelines and regulations.

### 2.3 Exposure - oral health problems

The standardised screening tool, the Revised Oral Assessment Guide-Jönköping (ROAG-J) [18,19] was used to assess oral health status. ROAG has previously been validated [20]. The tool relies on observations by trained non-dental health care personnel to evaluate the oral health status, and if indicated, the need for intervention. ROAG-J assesses nine items: voice, swallowing function, lips, mucus membranes, tongue, saliva, gums, teeth, and dentures. Each item is graded 1−3: score (1) healthy or normal condition, score (2) a moderate problem, and score (3) a severe problem. A referral to a dentist or physician is advised when one or more severe problems are observed. A score of 0 can also be used whenever the item is not applicable to assess, which applies to the items voice, swallowing function, gums, teeth, and dentures. Specifically, for the items gums and teeth, a score of 0 means that the person does not have teeth, and for dentures, a score of 0 means the person does not have dentures [18]. For further description of ROAG-J in HUNT4 70+ see Cetrelli et al. [21].

Studies that have used ROAG-J or ROAG have presented their results in various ways [[22], [23], [24]]. We chose to present the ROAG-J results as follows: (1) Dichotomous variables of each individual ROAG-J item: scores were grouped as no problem (score 1), or problem (score 2 and 3), score 0 was excluded. Scores 2 and 3 were grouped as they are both considered problems or deviations from a normal condition. (2) A continuous ROAG-J total score (range 9–27): the scores of all nine items were added up. Score 0 was treated as score 1 to preserve the sample size and to facilitate interpretation of the total score. The higher the score, the worse the oral health status. (3) An ordinal variable according to the severity of the ROAG-J scores, with three categories: no oral health problems (score 0 or 1 for all items), moderate oral health problem (at least one score 2, no score 3), and severe oral health problem (at least one score 3).

In addition to ROAG-J, the oral health assessment included information on the presence of teeth (yes/no), the number of teeth (more than six teeth in each jaw), and the use of dentures (full or partial in each jaw). We constructed an ordinal variable for the number of teeth with the categories: more than six teeth in both jaws, six or fewer teeth in one or both jaws, and edentulous. The use of full or partial dentures was used as a dichotomous variable (yes/no). Seventeen participants lacked information on placement and the number of teeth.

### 2.4 Outcome - malnutrition

We used three variables for malnutrition: body mass index (BMI), involuntary weight loss, and food intake. These are widely used in malnutrition screening and diagnostics [2,25]. For BMI, height and weight measurements were performed with Seca 813 scales and Seca 217 Stadiometer (Seca, Germany). Measurements were registered to the nearest centimetre and 0.1 kilogram (kg). BMI was calculated by dividing weight (kg) by the squared height in meters (kg/m^2^). For this study, BMI was dichotomized: <22 kg/m^2^ and ≥22 kg/m^2^, the age-specific cut-off for underweight among adults aged ≥70 years [25]. Data on involuntary weight loss (yes/no) were self-reported from questionnaires derived from the question “In the past six months, have you involuntarily lost more than five kilograms of body weight?”. Data on food intake was collected during the standardised interview. The question asked was “How would you describe your/his/her food intake the last four weeks?” with the response alternatives: more than usual, as usual, a bit less than usual (3/4), half of usual, and almost nothing. For this study, we combined the last two responses into ‘severely reduced food intake’.

From these variables, we constructed a primary outcome variable based on the work by Kolberg et al. [26]. Malnutrition, as a dichotomous variable (yes/no), was defined as present if at least one of three criteria was met: BMI <22 kg/m^2^, involuntary weight loss of >5 kg in the last six months, or a severely reduced food intake in the last four weeks.

### 2.5 Other variables and covariates

Age at participation was used as both a continuous and a categorical variable: 70−74, 75−79, 80−84, and ≥85 years. Sex was presented as men and women. Marital status was categorised into unmarried, married, widow(er), and separated/divorced. Sex and marital status were collected by HUNT from the National Population Register of Norway. Highest attained education was based on data from Statistics Norway [27] and categorised into: ≤10 years (primary school), 11−13 years (secondary school), and ≥14 years (college/university). Cognitive function, based on the Diagnostic and Statistical Manual of Mental Disorders, fifth edition (DSM-5) diagnostic criteria, was grouped into no cognitive impairment, mild cognitive impairment (MCI), dementia, and other reasons for cognitive impairment or unable to diagnose [28]. Self-perceived health, collected through a self-report questionnaire, was dichotomised into good and poor. Variables with missing observations were given a ‘unknown’ category.

### 2.6 Statistical analysis

General characteristics were presented for the total sample and stratified by the presence of oral health problems (according to the ROAG-J total score: no problem [score 9]/problems [score >9]). Descriptive results were presented with frequencies and proportions (%), and median with interquartile range (IQR) where appropriate.

The association between oral health problems and the prevalence of malnutrition was assessed using Poisson regression with robust error variance. This model has been shown to yield correct estimates when binary outcome variables are common [29]. All Poisson regression models were adjusted for potential confounders, including sex, age (categorical), education, cognitive function, marital status, and self-perceived health. Confounders were identified based on a priori knowledge and the literature [1,11,30,31]. Results of the regression analyses were presented as prevalence ratios (PR) with 95% confidence intervals (CI). P-for-trend was also presented for the association between the ordinal ROAG-J variable (treated as a continuous variable) and malnutrition.

To address potential bias due to missing observations for the covariates and number of teeth, we conducted additional analysis by applying multiple imputation by chained equations [32]. We assumed that missing at random was applicable to these variables. Twenty datasets were imputed. The Poisson models were then repeated using the Stata command *mi*, and the averaged estimates were given as inferential statistics (shown in supplementary file).

Data analyses were conducted using StataNow/SE version 19.5 (College Station, TX: StataCorp LLC).

## 3 Results

### 3.1 General characteristics

The majority of the 1571 participants were from the Trondheim 70+ cohort (73.5%) (Fig. 1) and younger than 80 years (61.0%) (Table 1). More than half of the study sample (57.5%) had at least one oral health problem. Compared to participants with no oral health problems, a larger proportion of participants with problems were older than 84 years, widow(er), had dementia, and had poor self-perceived health. More men than women had oral health problems. A larger proportion of participants who were visited at home or in nursing homes had oral health problems compared to participants examined at field stations (Table 1). Moderate problems (score 2) were more common than severe problems (score 3) for all items (Supplementary Table S1). Malnutrition was present in 17.8% (Table 3). More women than men met the criteria for malnutrition (21.4% vs. 12.8%) (Supplementary Table S2). Differences in general characteristics between the study sample and the excluded population are shown in Supplementary Table S3.

**Table 1:** General characteristics in total study sample (n = 1571) and stratified by oral health.

	Total n = 1571	Oral health^a^	
		No problem n = 667 (42.5%)	Problems n = 904 (57.5%)
Age			
Median (IQR)	76.9 (73.2−84.7)	75.2 (72.6−80.8)	78.9 (73.9−87.2)
Age groups, n (%)			
70−74 years	597 (38.0)	320 (48.0)	277 (30.6)
75−79 years	362 (23.0)	155 (23.2)	207 (22.9)
80−84 years	230 (14.6)	99 (14.8)	131 (14.5)
≥85 years	382 (24.3)	93 (13.9)	289 (32.0)
Sex, n (%)			
Women	905 (57.6)	406 (60.9)	499 (55.2)
Men	666 (42.4)	261 (39.1)	405 (44.8)
Marital status, n (%)			
Unmarried	78 (5.0)	25 (3.8)	53 (5.9)
Married	795 (50.6)	374 (56.1)	421 (46.6)
Widow(er)	505 (32.2)	174 (26.1)	331 (36.6)
Separated/Divorced	181 (11.5)	87 (13.0)	94 (10.4)
Unknown	12 (0.8)	7 (1.1)	5 (0.6)
Education, n (%)			
Primary (≤10 years)	323 (20.6)	109 (16.3)	214 (23.7)
Secondary (11−13 years)	705 (44.9)	286 (42.9)	419 (46.4)
College/University (≥14 years)	543 (34.6)	272 (40.8)	271 (30.0)
Cognitive function, n (%)			
No cognitive impairment	703 (44.8)	366 (54.9)	337 (37.3)
MCI	520 (33.1)	208 (31.2)	312 (34.5)
Dementia	328 (20.9)	86 (12.9)	242 (26.8)
Unknown^b^	20 (1.3)	7 (1.1)	13 (1.4)
Self-perceived health, n (%)			
Good	1041 (66.3)	498 (74.7)	543 (60.1)
Poor	483 (30.7)	151 (22.6)	332 (36.7)
Unknown	47 (3.0)	18 (2.7)	29 (3.2)
Test location, n (%)			
Field station	1049 (66.8)	527 (79.0)	522 (57.7)
Home visit	409 (26.0)	109 (16.3)	300 (33.2)
Nursing home	113 (7.2)	31 (4.7)	82 (9.1)

BMI: body mass index, IQR: interquartile range, MCI: mild cognitive impairment, SD: standard deviation, ROAG-J: Revised Oral Assessment Guide – Jönköping.

a No problem: ROAG-J total score = 9 (only score 0 or 1), Problems: ROAG-J total score >9 (at least one score 2 or 3).

b Other cause of cognitive impairment or not able to diagnose or missing observation.

### 3.2 Association between oral health problems and malnutrition

A crude higher prevalence of malnutrition was found for several ROAG-J item problems. However, in the adjusted models, a higher prevalence was only found for problems with voice, lips, and dentures (Table 2). Furthermore, the prevalence of malnutrition increased with every increase in ROAG-J total score (adjusted PR 1.02, 95% CI 1.01–1.03) and across problem severity (p-for-trend <0.01) (Table 3). Among those with at least one severe oral health problem, the prevalence of malnutrition was 9% higher compared to those without any oral health problems (adjusted PR 1.09, 95% CI 1.03–1.15). A crude higher prevalence of malnutrition was seen in those with six or fewer teeth in one or both jaws, the edentulous, and those using dentures, although these differences in prevalence were not statistically significant in the adjusted models (Table 3). Additional analyses based on the imputed data for missing values of the covariates, including the number of teeth, yielded similar results (Supplementary Table S4 compared to Table S3).

**Table 2:** Association between individual ROAG-J items (dichotomous) and prevalence of malnutrition.

ROAG-J^a^		Total n	Malnutrition^b^ n (%)	Crude PR (95% CI)	Adjusted^c^ PR (95% CI)
Voice (n = 1571)	No problem	1359	215 (15.8)	Ref.	Ref.
	Problem	212	64 (30.2)	1.12 (1.07, 1.18)	1.10 (1.05, 1.16)
Swallowing function (n = 1569)	No problem	1426	244 (17.1)	Ref.	Ref.
	Problem	143	34 (23.8)	1.06 (1.00, 1.12)	1.03 (0.97, 1.09)
Lips (n = 1571)	No problem	1382	232 (16.8)	Ref.	Ref.
	Problem	189	47 (24.9)	1.07 (1.01, 1.13)	1.06 (1.01, 1.11)
Mucus Membranes (n = 1571)	No problem	1400	248 (17.7)	Ref.	Ref.
	Problem	171	31 (18.1)	1.00 (0.95, 1.06)	1.01 (0.96, 1.06)
Tongue (n = 1571)	No problem	1325	224 (16.9)	Ref.	Ref.
	Problem	246	55 (22.4)	1.05 (1.00, 1.10)	1.04 (1.00, 1.09)
Saliva (n = 1571)	No problem	1519	267 (17.6)	Ref.	Ref.
	Problem	52	12 (23.1)	1.05 (0.95, 1.15)	1.01 (0.92, 1.11)
Gums (n = 1347)	No problem	1267	202 (15.9)	Ref.	Ref.
	Problem	80	19 (23.8)	1.07 (0.99, 1.15)	1.05 (0.97, 1.14)
Teeth (n = 1347)	No problem	926	150 (16.2)	Ref.	Ref.
	Problem	421	71 (16.9)	1.01 (0.97, 1.04)	0.99 (0.95, 1.03)
Dentures (n = 550)	No problem	369	65 (17.6)	Ref.	Ref.
	Problem	181	57 (31.5)	1.12 (1.05, 1.19)	1.09 (1.01, 1.16)

BMI: body mass index, CI: confidence interval, Ref.: reference, ROAG-J: Revised Oral Assessment Guide – Jönköping, PR: prevalence ratio.

a ROAG-J items presented dichotomized (no problem: score 1, problem: score 2 or 3), score 0 excluded for voice (n = 0), swallowing function (n = 2), gums (n = 224), teeth (n = 224) and dentures (n = 1021).

b Malnutrition present if any of the following three criteria are met: BMI < 22 kg/m^2^, involuntary weight loss of >5 kg last six months, or severely reduced food intake the last four weeks.

c Adjusted for sex, age, education, cognitive function, marital status, and self-perceived health.

**Table 3:** Association between oral health problems and the prevalence of malnutrition.

	Total n	Malnutrition^a^ n (%)	Crude PR (95% CI)	Adjusted^b^ PR (95% CI)
Total	1571	279 (17.8)		
ROAG-J total score				
Median (IQR)	10 (9−11)	10 (9−12)	1.03 (1.01, 1.04)	1.02 (1.01, 1.03)
ROAG-J ordinal				
No problem^c^	667	95 (14.2)	Ref.	Ref.
Moderate problem^d^	726	137 (18.9)	1.04 (1.01, 1.08)	1.03 (1.00, 1.07)
Severe problem^e^	178	47 (26.4)	1.11 (1.05, 1.17)	1.09 (1.03, 1.15)
p-trend				<0.01
Number of teeth	**n = 1554**^f^	**n = 275 (17.7)**		
>6 in both jaws	1141	171 (15.0)	Ref.	Ref.
≤6 in one or both jaws	189	46 (24.3)	1.08 (1.03, 1.14)	1.05 (0.99, 1.11)
Edentulous	224	58 (25.9)	1.09 (1.04, 1.15)	1.04 (0.98, 1.10)
Full or partial dentures				
No	1021	157 (15.4)	Ref.	Ref.
Yes	550	122 (22.2)	1.06 (1.02, 1.10)	1.02 (0.98, 1.05)

BMI: body mass index, CI: confidence interval, IQR: interquartile range, Ref: reference, ROAG-J: Revised Oral Assessment Guide-Jönköping, PR: prevalence ratio.

a Malnutrition present if any of the following three criteria are met: BMI < 22 kg/m^2^, involuntary weight loss of >5 kg last six months, or severely reduced food intake the last four weeks.

b Adjusted for sex, age, education, cognitive function, marital status, and self-perceived health.

c Score 0 or 1 for all ROAG-J items.

d At least one ROAG-J item with score 2 (moderate problem), but no score 3 (severe problem).

e At least one ROAG-J item with score 3 (severe problem).

f Missing information on number of teeth n = 17.

## 4 Discussion

In this population-based sample of Norwegian older adults, an increase in the ROAG-J total score, having at least one severe oral health problem, and problems with voice, lips, and dentures were associated with a higher prevalence of malnutrition. We also found an indication that the number of teeth and the use of dentures were not associated with a higher prevalence of malnutrition, although the crude prevalences suggest otherwise.

We found a positive association between ROAG-J total score and the prevalence of malnutrition. A similar stepwise increase in oral symptom burden (dry mouth, chewing problems, and swallowing problems) and malnutrition, was found in the study by Lindroos et al. [33], who investigated Finnish older adults in institutional settings. Higher occurrence of oral health problems (per ROAG/ROAG-J) among those being underweight and at risk of malnutrition has also been found among Swedish nursing home residents and older adults in short-term care [23,34,35]. Moreover, we found that older adults with severe oral health problems had a higher prevalence of malnutrition than those without oral health problems. A similar categorisation of problem severities was reported in a study investigating hospitalized older adults, although in this study the proportion of malnutrition was larger among those with moderate problems rather than severe problems (per ROAG) [22]. The same study also reported that those with at least one moderate or severe oral health problem had higher odds of malnutrition risk and frailty [22].

Furthermore, problems with voice, lips, and dentures were separately associated with a higher prevalence of malnutrition. A similar finding was reported by Lindmark et al. [23], who found an association between problems with voice and swallowing function and the risk of malnutrition and malnutrition among Swedish care-needing older adults. A study including independent and care-dependent older adults aged ≥65 years in the Netherlands, adjusted for age and gender, suggested that broken teeth, oral pain/discomfort, chewing and swallowing problems, adaptation of food, and coughing were associated with involuntary weight loss or low BMI [36]. Although participants with swallowing and tongue problems had slightly higher prevalences of malnutrition in our study, there was no statistically significant association after adjusting for confounders. The observed associations for individual items should, however, be interpreted with caution as we did not adjust for multiple comparisons. In the context of ROAG-J, oral health problems can indicate a generally poor health condition, oral dryness, poor oral hygiene, pain, ulcers and blisters, coatings, missing and broken teeth, and altered fit and function or non-use of dentures [18]. It could also indicate oral symptoms of nutritional deficiencies and malnutrition, underscoring a possible bidirectional association [18]. Diet, including surpluses and deficits of nutrients, has been linked to oral disease and oral symptoms [37,38].

Having problems with remaining teeth (i.e., having food debris, coatings on some or all teeth, or severely broken teeth) was not associated with malnutrition in our study. When we consider the findings for the number of teeth, the results, despite attenuation in the adjusted model, indicate a trend toward higher prevalence of malnutrition among those with six or fewer teeth in one or both jaws and the edentulous. The reporting of tooth count varies greatly between studies, but recent systematic reviews with meta-analyses suggest that having a reduced number of teeth, but not necessarily edentulousness or denture use, is associated with risk of malnutrition and malnutrition [9,10]. It is important to emphasise that the relationship with teeth is likely affected by the timing of the assessment and the presence of dentures. A Japanese longitudinal study, which adjusted for denture use in their analyses, found that compared to those with self-reported ≥20 teeth, those with 10–19 and 0–9 teeth had a higher risk of >5% weight loss over three years among independent older adults aged ≥65 years [12]. Although in the Longitudinal Ageing Study Amsterdam (baseline age 55–80) [13], self-reported edentulism, having at least one jaw with one to seven teeth, and denture use were not associated with the incidence of low BMI and/or ≥5% weight loss the last six months over nine years.

Similarly, as for the number of teeth, denture wearers had a higher crude prevalence of malnutrition, but this association was not statistically significant after adjusting for confounders. However, the condition and function of the dentures, as reflected by the ROAG-J item, remained associated with malnutrition when adjusting for confounders. This suggests that denture wearers, particularly those with denture problems, may be at risk of malnutrition. A similar observation of a larger crude proportion of participants with denture problems (per ROAG-J) among those at risk and malnourished was seen among care-needing older adults in Lindmark et al. [23]. Some studies suggest that full dentures and self-reported poorly fitting dentures are associated with a lower intake of certain foods and nutrients [39,40]. However, dentures can also positively affect protein intake among individuals with few to no teeth [41]. It is therefore possible that long-term problems with dentures, including non-use, could impact body weight and risk of malnutrition over time. In two longitudinal studies (baseline age ≥60 and 55–80), self-reported difficulties with chewing hard foods and chewing pain were associated with increased odds and risk of involuntary weight loss and/or low BMI and malnutrition among community dwellers [13,42]. This underscores the importance of addressing dentition-related problems early and the need for denture maintenance. Health care workers should be aware that denture wearers, especially those experiencing impaired denture function and condition, could be at risk of developing malnutrition or be malnourished.

In our study, 57.5% of participants had at least one oral health problem, and 17.8% were malnourished. These prevalences are high and similar to those reported in care settings and possibly for combined populations of independent and care-dependent older adults [2,34,43]. Considering this and the findings from our previous prevalence studies on oral health problems and malnutrition [21,26], oral health problems should receive greater attention in malnutrition assessment. Similarly, the risk of malnutrition should be considered when problems are identified during oral health examinations. Multidisciplinary teams and health care workers would benefit from receiving training in both oral health and malnutrition screening to aid faster identification and initiation of appropriate measures. Several studies also suggest that special attention should be given to care-dependent community-dwelling older adults, as their oral health is poor [37].

The current study has several strengths. The sample population was large and included both care-dependent and independent older adults. The comprehensive assessment of oral health enabled us to determine the extent of oral health problems and differentiate between the severity and types of problems. The malnutrition definition we used is broad and includes only items directly related to malnutrition. Furthermore, the definition is sufficiently specific to identify individuals who might otherwise be lost in other composite definitions. This study also has some limitations. The cross-sectional design prevents the interpretation of causality. This is further complicated by the possible bidirectional relationship between oral health problems and malnutrition. Additionally, selection bias should be considered given the undercoverage of participants in HUNT4 70+ (Fig. 1 and Supplementary Table S3). The excluded population was slightly younger, fewer had diagnosed dementia, and a larger proportion reported poor self-perceived health. This may have impacted the results, however, the direction and magnitude of its impact on the prevalence estimates and observed associations were difficult to assess. Furthermore, as our results are derived from a homogeneous Norwegian population, the findings may be transferable to other Western countries with similar health care systems, but possibly not directly transferable to populations with different ethnic backgrounds. Although we adjusted our analyses for many potential confounders, residual confounding is possible. Lastly, recall bias is also a possibility, as some covariates and components of the outcome were based on self-reported data.

The identified associations of this study have implications for future research and clinical practice. Prospective studies are needed to support causality in either direction of the association between oral health and malnutrition. Preserving adequate oral health and avoiding malnutrition is part of healthy ageing [44]. Changes in anthropometric measures, food intake, and oral health should therefore not be dismissed. Health care workers should be aware of the association between poor oral health and malnutrition, and that both have been linked to poor quality of life and mortality among older adults [[45], [46], [47], [48]]. Screening can be an effective way of identifying individuals at risk or in an active poor state [49]. In addition to educating health care workers, greater emphasis on integration in health policy and interdisciplinary cooperation across sectors may further strengthen efforts to address oral health problems and malnutrition in older adults globally [50,51]. Ripple effects may also be expected as treating malnutrition and oral health problems reduces healthcare costs and risk of adverse health outcomes [52,53].

## 5 Conclusion

This study showed that poor oral health status and having one or more severe oral health problems were associated with malnutrition measured by low BMI, involuntary weight loss, and/or severely reduced food intake among older adults. Further research should prospectively assess the relationship between the condition and function of oral health and malnutrition. Interdisciplinary collaboration on oral health assessment in malnutrition prevention and treatment, and vice versa, should be prioritised and would likely improve the quality of care for older adults.

## Author contributions

According to the CRediT roles (http://credit.niso.org/) the authors contributed with the following: Investigation by MK, LMB, TNF, HKS, YQS, HH, and SHC. Project administration by MK and SHC. Funding acquisition by MK and WMT. Conceptualization by MK, YQS, WMT, IP, SHC, TNF, and HH. Data Curation by MK, LMB, and SHC. Methodology by SHC, YQS, MK, IP, and WMT. Formal analysis by SHC. Supervision by MK, YQS, WMT, and IP. Writing – original draft by SHC. Visualization by SHC. Writing – review & editing by SHC, YQS, MK, WMT, IP, LMB, HH, TNF, and HKS.

## Declaration of Generative AI and AI-assisted technologies in the writing process

During literature searches for this manuscript, the free open-source tool Inciteful (https://inciteful.xyz/) was used as a supplement to academic search engines for identifying relevant literature. Papers identified from searches on this website were reviewed in full by the first author (SHC).

## Funding

This manuscript is to be part of the Ph.D. thesis of SHC. This Ph.D. project received funding from the Faculty of Medicine and Health Sciences at The Norwegian University of Science and Technology (NTNU). This Ph.D. project is also part of the DigiFrailCare project which received funding from NTNU Health (https://www.ntnu.edu/health/digifrailcare).

NTNU provided open access funding.

YQS was supported by a research grant from the Liaison Committee for Education, Research and Innovation in Central Norway (project ID 2018/42794).

The Norwegian Directorate of Health provided financial support through their support of research at the Norwegian Centres for Oral Health Services and Research (https://www.helsedirektoratet.no/tilskudd/etablering-og-drift-av-regionale-odontologiske-kompetansesentre).

## Data availability statement

Data used in this study cannot be deposited in open repositories due to the protection of participant privacy and the rights of researchers using HUNT data. With approval of application to HUNT Research Centre the exact dataset used in this study can be obtained.

## Declaration of competing interest

The authors have no conflict of interest to declare.

## References

[1] Dent E., Wright O.R.L., Woo J., Hoogendijk E.O. (2023). Malnutrition in older adults. Lancet.

[2] Wolters M., Volkert D., Streicher M., Kiesswetter E., Torbahn G., O’Connor E.M. (2019). Prevalence of malnutrition using harmonized definitions in older adults from different settings – A MaNuEL study. Clin Nutr.

[3] United Nations. World Population Prospects 2024: Summary of Results. UN DESA/POP/2024/TR/NO. 9. Accessed February 10, 2026. https://population.un.org/wpp/assets/Files/WPP2024_Summary-of-Results.pdf.

[4] Glick M., Williams D.M., Kleinman D.V., Vujicic M., Watt R.G., Weyant R.J. (Dec 2017). A new definition for oral health developed by the FDI World Dental Federation opens the door to a universal definition of oral health. J Public Health Dent.

[5] Volkert D., Kiesswetter E., Cederholm T., Donini L.M., Eglseer D., Norman K. (Jan–Dec 2019). Development of a model on determinants of malnutrition in aged persons: a MaNuEL project. Gerontol Geriatr Med.

[6] Volkert D., Beck A.M., Cederholm T., Cruz-Jentoft A., Hooper L., Kiesswetter E. (Apr 2022). ESPEN practical guideline: clinical nutrition and hydration in geriatrics. Clin Nutr.

[7] Kiesswetter E., Poggiogalle E., Migliaccio S., Donini L.M., Sulmont-Rossé C., Feart C. (Jul 2018). Functional determinants of dietary intake in community-dwelling older adults: a DEDIPAC (DEterminants of DIet and Physical ACtivity) systematic literature review. Public Health Nutr.

[8] Nitsuwat S., Webster J., Sarkar A., Cade J. (Mar 1 2025). The association of oral processing factors and nutrient intake in community-dwelling older adults: a systematic review and meta-analysis. Nutr Rev.

[9] Toniazzo M.P., Amorim P.S., Muniz F., Weidlich P. (Jun 2018). Relationship of nutritional status and oral health in elderly: systematic review with meta-analysis. Clin Nutr.

[10] Zelig R., Goldstein S., Touger-Decker R., Firestone E., Golden A., Johnson Z. (Jan 2022). Tooth loss and nutritional status in older adults: a systematic review and meta-analysis. JDR Clin Trans Res.

[11] O’Keeffe M., Kelly M., O’Herlihy E., O’Toole P.W., Kearney P.M., Timmons S. (Dec 2019). Potentially modifiable determinants of malnutrition in older adults: a systematic review. Clin Nutr.

[12] Shiota C., Kusama T., Takeuchi K., Kiuchi S., Osaka K. (2023). Oral hypofunction and risk of weight change among independent older adults. Nutrients.

[13] Kiesswetter E., Hengeveld L.M., Keijser B.J., Volkert D., Visser M. (Jun 2019). Oral health determinants of incident malnutrition in community-dwelling older adults. J Dent.

[14] Bakker M.H., Vissink A., Spoorenberg S.L.W., Jager-Wittenaar H., Wynia K., Visser A. (Dec 12 2018). Are Edentulousness, oral health problems and poor health-related quality of life associated with malnutrition in community-dwelling elderly (Aged 75 Years and Over)? A cross-sectional study. Nutrients.

[15] Everaars B., Weening-Verbree L.F., Jerković-Ćosić K., Schoonmade L., Bleijenberg N., de Wit N.J. (Jan 3 2020). Measurement properties of oral health assessments for non-dental healthcare professionals in older people: a systematic review. BMC Geriatr.

[16] Åsvold B.O., Langhammer A., Rehn T.A., Kjelvik G., Grøntvedt T.V., Sørgjerd E.P. (2022). Cohort profile update: the HUNT study, Norway. Int J Epidemiol.

[17] Skjellegrind H.K., Thingstad P., Gjøra L., Kolberg M., Kjelvik G., Ernstsen L. (Feb 18 2026). Cohort profile update: HUN 70+. Int J Epidemiol.

[18] Senior Alert. Manual - för riskbedömningsinstrumentet ROAG Revised Oral Assessment Guide (ROAG) och Munvårdsåtegärder - Förslag vid en eller flera 2:or och/eller 3:or i ROAG-J. Internet. Senior Alert. Accessed February 10 2026. https://www.senioralert.se/media/gyjl5otu/manual_och_atgarder_roag_version2.pdf.

[19] Andersson P., Hallberg I.R., Renvert S. (Sep–Oct 2002). Inter-rater reliability of an oral assessment guide for elderly patients residing in a rehabilitation ward. Spec Care Dentist.

[20] Ribeiro M.T., Ferreira R.C., Vargas A.M., Ferreira e Ferreira E. (Jun 2014). Validity and reproducibility of the revised oral assessment guide applied by community health workers. Gerodontology.

[21] Cetrelli S.H., Sun Y.-Q., Thorstensen W.M., Høvik H., Gjøra L., Berg L.M. (Sep 30 2025). Prevalence of oral health problems in a Norwegian older adult population: the HUNT study. BMC Oral Health.

[22] Chew J., Chia J.Q., Kyaw K.K., Fu J.K., Ang J., Lim Y.P. (2023). Association of oral health with frailty, malnutrition risk and functional decline in hospitalized older adults: a cross-sectional study. J Frailty Aging.

[23] Lindmark U., Jansson H., Lannering C., Johansson L. (Mar 2018). Oral health matters for the nutritional status of older persons-A population-based study. J Clin Nurs.

[24] Mostad I.L., Reinan T.K., Halgunset J., Thoresen L., Feuerherm A.J., Kolberg M. (2023). Oral health problems are associated with malnutrition in hospitalised adult patients. Clin Nutr ESPEN.

[25] Cederholm T., Jensen G.L., Correia M., Gonzalez M.C., Fukushima R., Higashiguchi T. (Feb 2019). GLIM criteria for the diagnosis of malnutrition - A consensus report from the global clinical nutrition community. Clin Nutr.

[26] Kolberg M., Paur I., Sun Y.-Q., Gjøra L., Skjellegrind H.K., Thingstad P. (2023). Prevalence of malnutrition among older adults in a population-based Study - the HUNT Study. Clin Nutr ESPEN.

[27] Statistics Norway. Dokumentasjonsartikkel, Nasjonal utdanningsdatabase. Internet. Statistics Norway. Updated 18 May 2020. Accessed February 20, 2026. https://www.ssb.no/data-til-forskning/utlan-av-data-til-forskere/variabellister/utdanning/nasjonal-utdanningsdatabase.

[28] Gjøra L., Strand B.H., Bergh S., Borza T., Brækhus A., Engedal K. (2021). Current and future prevalence estimates of mild cognitive impairment, dementia, and its subtypes in a population-based sample of people 70 years and older in Norway: the HUNT study. J Alzheimers Dis.

[29] Barros A.J., Hirakata V.N. (Oct 20 2003). Alternatives for logistic regression in cross-sectional studies: an empirical comparison of models that directly estimate the prevalence ratio. BMC Med Res Methodol.

[30] Streicher M., van Zwienen-Pot J., Bardon L., Nagel G., Teh R., Meisinger C. (Dec 2018). Determinants of incident malnutrition in community-dwelling older adults: a MaNuEL Multicohort Meta-Analysis. J Am Geriatr Soc.

[31] Tsakos G., Watt R.G., Guarnizo-Herreño C.C. (2023). Reflections on oral health inequalities: theories, pathways and next steps for research priorities. Community Dent Oral Epidemiol.

[32] White I.R., Royston P., Wood A.M. (2011). Multiple imputation using chained equations: issues and guidance for practice. Stat Med.

[33] Lindroos E.K., Saarela R.K.T., Suominen M.H., Muurinen S., Soini H., Kautiainen H. (May 2019). Burden of oral symptoms and its associations with nutrition, well-being, and survival among nursing home residents. J Am Med Dir Assoc.

[34] Bellander L., Andersson P., Nordvall D., Hägglin C. (May 2021). Oral health among older adults in nursing homes: a survey in a national quality register, the Senior Alert. Nurs Open.

[35] Lindqvist S., Olai L., Hägglund P. (Jun 2 2024). Factors associated with malnutrition among older people in Swedish short-term care: poor oral health, dysphagia and mortality. Int J Dent Hyg.

[36] Hollaar V., de van der Schueren M., Haverkort E., Everaars B., Borkent J., Jerković-Ćosić K. (2025). Associations between problems in oral health, oral function and malnutrition in older people: results from three databases. Int J Dental Hygiene.

[37] Chan A.K.Y., Tsang Y.C., Jiang C.M., Leung K.C.M., Lo E.C.M., Chu C.H. (Sep 19 2023). Diet, nutrition, and oral health in older adults: a review of the literature. Dent J (Basel).

[38] Beavan S., Patel N., Dave M. (2026). Nutritional deficiencies and their oral manifestations. BDJ Team.

[39] Iwasaki M., Taylor G.W., Manz M.C., Yoshihara A., Sato M., Muramatsu K. (Oct 2014). Oral health status: relationship to nutrient and food intake among 80-year-old Japanese adults. Community Dent Oral Epidemiol.

[40] Jauhiainen L., Männistö S., Ylöstalo P., Vehkalahti M., Nordblad A., Turunen A.W. (2017). Food consumption and nutrient intake in relation to denture use in 55- to 84-year-old men and women -results of a population based survey. J Nutr Health Aging.

[41] Kusama T., Takeuchi K., Kiuchi S., Aida J., Hikichi H., Sasaki S. (Nov 2023). Dental prosthesis use is associated with higher protein intake among older adults with tooth loss. J Oral Rehabil.

[42] Lexomboon D., Kumar A., Freyland S., Xu W., Sandborgh-Englund G. (Jun 2025). Is poor chewing ability a risk factor for malnutrition? A six-year longitudinal study of older adults in Sweden. J Nutr Health Aging.

[43] Henni S.H., Skudutyte-Rysstad R., Ansteinsson V., Hellesø R., Hovden E.A.S. (2023). Oral health and oral health-related quality of life among older adults receiving home health care services: a scoping review. Gerodontology.

[44] World Health Organization. World report on ageing and health. Internet. WHO. Accessed July 7, 2026. https://iris.who.int/handle/10665/186463.

[45] Chen K.Y., Lin S.Y., Weng S.C., Lee Y.S., Kuo F.H., Yeh Y.H. (Jun 4 2025). Using revised oral assessment guide to assess association between oral health and mortality in older inpatients: a single-center observational cohort study. BMC Oral Health.

[46] Lindmark U., Ernsth Bravell M., Johansson L., Finkel D. (Jun 2021). Oral health is essential for quality of life in older adults: a Swedish National Quality Register Study. Gerodontology.

[47] Rodríguez-Mañas L., Rodríguez-Sánchez B., Carnicero J.A., Rueda R., García-Garcia F.J., Pereira S.L. (Mar 2021). Impact of nutritional status according to GLIM criteria on the risk of incident frailty and mortality in community-dwelling older adults. Clin Nutr.

[48] Kvamme J.M., Olsen J.A., Florholmen J., Jacobsen B.K. (May 2011). Risk of malnutrition and health-related quality of life in community-living elderly men and women: the Tromsø study. Qual Life Res.

[49] World Health Organization. Screening. Internet. WHO. Accessed July 4 2026. https://www.who.int/europe/teams/ncd-management/screening.

[50] Dhanapriyanka M., Pritchard L., Zamora A., Torwane N., Schuch H.S., Ha D.H. (Apr 27 2026). Integrating oral health in general health national policy frameworks for older adults: scoping review. Gerodontology.

[51] Volkert D., Visser M., Corish C.A., Geisler C., de Groot L., Cruz-Jentoft A.J. (Feb 2020). Joint action malnutrition in the elderly (MaNuEL) knowledge hub: summary of project findings. Eur Geriatr Med.

[52] Toulson Davisson Correia M.I., Castro M., de Oliveira Toledo D., Farah D., Sansone D., de Morais Andrade T.R. (Sep 2021). Nutrition therapy cost-effectiveness model indicating how nutrition may contribute to the efficiency and financial sustainability of the health systems. JPEN J Parenter Enteral Nutr.

[53] Münzenmayer M.A., Mariño R., Hsueh A. (Jun 2019). Cost-effectiveness of professional oral health care in Australian residential aged care facilities. Gerodontology.

