# Supplementary material for: The association between oral health problems and malnutrition in a population-based sample of older adults – The HUNT Study
Source: J Nutr Health Aging. 2026 Aug 7;30(9):100935. doi: 10.1016/j.jnha.2026.100935 (PMC13475336; doi:10.1016/j.jnha.2026.100935)
Supplement: Supplementary file 1 [file mmc1.docx]

Supplementary table 1. Distribution of participants with the score 0-3 for each ROAG-J item (n=1571)

|  | **Score 0** | **Score 1** | **Score 2** | **Score 3** |
| --- | --- | --- | --- | --- |
| **Voice, n (%)** | 0 (0) | 1359 (86.5) | 205 (13.1) | 7 (0.5) |
| **Swallowing function, n (%)** | 2 (0.1) | 1426 (90.8) | 140 (8.9) | 3 (0.2) |
| **Lips, n (%)** |  | 1382 (88.0) | 184 (11.7) | 5 (0.3) |
| **Mucus membranes, n (%)** |  | 1400 (89.1) | 100 (6.4) | 71 (4.5) |
| **Tongue, n (%)** |  | 1325 (84.3) | 235 (15.0) | 11 (0.7) |
| **Saliva, n (%)** |  | 1519 (96.7) | 52 (3.3) | 0 (0) |
| **Gums, n (%)** | 224 (14.3) | 1267 (80.7) | 75 (4.8) | 5 (0.3) |
| **Teeth, n (%)** | 224 (14.3) | 926 (58.9) | 382 (24.3) | 39 (2.5) |
| **Dentures, n (%)** | 1021 (65.0) | 369 (23.5) | 129 (8.2) | 52 (3.3) |
|  |  |  |  |  |

ROAG-J: Revised Oral Assessment Guide – Jönköping.

Score 0: not applicable

Score 1: normal or healthy condition

Score 2: moderate problem or deviation

Score 3: severe problem or deviation

Supplementary table 2. Malnutrition variables and malnutrition in the total study sample, stratified by sex

|  | Total  n=1571 | Sex | |  |
| --- | --- | --- | --- | --- |
|  |  | Women | Men | |
|  |  | n=905 (57.6%) | n=666  (42.4%) | |
| **BMI, n (%)** |  |  |  | |
| ≥22 kg/m^2^ | 1386 (88.2) | 771 (85.2) | 615 (92.3) | |
| <22 kg/m^2^ | 185 (11.8) | 134 (14.8) | 51 (7.7) | |
| **Involuntary weight loss >5 kg last six months,**  **n (%)** |  |  |  | |
| No | 1467 (93.4) | 838 (92.6) | 629 (94.4) | |
| Yes | 104 (6.6) | 67 (7.4) | 37 (5.6) | |
| **Food intake last four weeks, n (%)** |  |  |  | |
| More than usual | 56 (3.6) | 33 (3.7) | 23 (3.5) | |
| As usual | 1363 (86.8) | 764 (84.4) | 599 (89.9) | |
| A bit less than usual | 123 (7.8) | 84 (9.3) | 39 (5.9) | |
| Half of usual | 24 (1.5) | 20 (2.2) | 4 (0.6) | |
| Almost nothing | 5 (0.3) | 4 (0.4) | 1 (0.2) | |
|  |  |  |  | |
| **Malnutrition^a^, n (%)** |  |  |  | |
| No | 1292 (82.2) | 711 (78.6) | 581 (87.2) | |
| Yes | 279 (17.8) | 194 (21.4) | 85 (12.8) | |
|  |  |  |  | |

BMI: body mass index

^a^ Yes if any of the following criteria are met: BMI < 22 kg/m^2^, involuntary weight loss of >5 kg last six months, or severely reduced food intake the last four weeks.

Supplementary table 3. General characteristics of the current study sample and excluded participants in HUNT4 70+

|  | | Study sample  n=1571 | Excluded |
| --- | --- | --- | --- |
|  |  |  | n=10128 |
| **Age** | |  |  |
| Median (IQR) | | 76.9 (73.2-84.7) | 76.3 (72.8-81.8) |
| **Age groups, n (%)** | |  |  |
| 70-74 years | | 597 (38.0) | 4197 (41.4) |
| 75-79 years | | 362 (23.0) | 2684 (26.5) |
| 80-84 years | | 230 (14.6) | 1645 (16.2) |
| ≥85 years | | 382 (24.3) | 1602 (15.8) |
| **Sex, n (%)** | |  |  |
| Women | | 905 (57.6) | 5523 (54.5) |
| Men | | 666 (42.4) | 4605 (45.5) |
| **Marital status, n (%)** | |  |  |
| Unmarried | | 78 (5.0) | 389 (3.8) |
| Married | | 795 (50.6) | 5736 (56.6) |
| Widow(er) | | 505 (32.2) | 2401 (23.7) |
| Separated/Divorced | | 181 (11.5) | 914 (9.0) |
| Unknown | | 12 (0.8) | 688 (6.8) |
| **Education, n (%)** | |  |  |
| Primary (≤10 years) | | 323 (20.6) | 2564 (25.3) |
| Secondary (11-13 years) | | 705 (44.9) | 5403 (53.4) |
| College/University (≥14 years) | | 543 (34.6) | 2151 (21.2) |
| Unknown | | 0 (0.0) | 10 (0.1) |
| **Cognitive function, n (%)** | |  |  |
| No cognitive impairment | | 703 (44.8) | 4914 (48.5) |
| MCI | | 520 (33.1) | 3457 (34.1) |
| Dementia | | 328 (20.9) | 1566 (15.5) |
| Unknown^a^ | | 20 (1.3) | 191 (1.9) |
| **Self-perceived health, n (%)** | |  |  |
| Good | | 1041 (66.3) | 5699 (56.3) |
| Poor | | 483 (30.7) | 3290 (32.5) |
| Unknown | | 47 (3.0) | 1139 (11.3) |
| **Test location, n (%)** | |  |  |
| Field station | | 1049 (66.8) | 8760 (86.5) |
| Home visit | | 409 (26.0) | 776 (7.7) |
| Nursing home | | 113 (7.2) | 592 (5.9) |
|  |  | |  |

IQR: interquartile range, MCI: mild cognitive impairment, SD: standard deviation.

^a^ Other cause of cognitive impairment or not able to diagnose or missing observation.

Supplementary table 4. Analyses for the association between oral health problems and malnutrition after applying multiple imputation by chained equations* (n=1571)

|  | Total  n | Malnutrition^a^  (%) | Crude  PR (95% CI) | Adjusted^b^  PR (95% CI) |
| --- | --- | --- | --- | --- |
|  |  |  |  |  |
| Total | 1571 | 17.8 |  |  |
| **ROAG-J total score** |  |  |  |  |
| Median (IQR) | 10 (9-11) | 10 (9-12) | 1.03 (1.01, 1.04) | 1.02 (1.01, 1.03) |
| **ROAG-J ordinal** |  |  |  |  |
| No problem^c^ | 667 | 14.2 | Ref. | Ref. |
| Moderate problem^d^ | 726 | 18.9 | 1.04 (1.01, 1.08) | 1.03 (1.00, 1.07) |
| Severe problem^e^ | 178 | 26.4 | 1.11 (1.05, 1.17) | 1.09 (1.03, 1.15) |
| p-trend |  |  |  | <0.01 |
| **Number of teeth (%)^f^** |  |  |  |  |
| >6 in both jaws | 1151 (73.3) | 15.0 | Ref. | Ref. |
| ≤6 in one or both jaws | 192 (12.2) | 24.5 | 1.08 (1.03, 1.14) | 1.05 (0.99, 1.11) |
| Edentulous | 228 (14.5) | 25.9 | 1.09 (1.04, 1.15) | 1.04 (0.98, 1.10) |
| **Full or partial dentures** |  |  |  |  |
| No | 1021 | 15.4 | Ref. | Ref. |
| Yes | 550 | 22.2 | 1.06 (1.02, 1.10) | 1.02 (0.98, 1.05) |
|  |  |  |  |  |

BMI: body mass index, CI: confidence interval. IQR: Interquartile range. Ref: reference. ROAG-J: Revised Oral Assessment Guide-Jönköping. PR: prevalence ratio.

^a^ Malnutrition present if any of the following three criteria are met: BMI <22 kg/m^2^, involuntary weight loss of >5 kg last six months, or severely reduced food intake the last four weeks.

^b^ Adjusted for sex, age, education, cognitive function, marital status, and self-perceived health.

^c^ Score 0 and 1 for all items (not applicable and normal or healthy)

^d^ At least one ROAG-J item with score 2 (moderate oral health problem), but no score 3 (severe oral health problem).

^e^ At least one ROAG-J item with score 3 (severe oral health problem).

^f^ Data imputed for 17 participants, the column total and malnutrition show the estimated total and stratified proportions of malnutrition for each category.

*Multiple imputation by chained equations for confounders and number of teeth.
